# The impact of lockdown during SARS-CoV-2 outbreak on behavioral and psychological symptoms of dementia

**DOI:** 10.1007/s10072-020-05035-8

**Published:** 2021-01-14

**Authors:** Arianna Manini, Michela Brambilla, Laura Maggiore, Simone Pomati, Leonardo Pantoni

**Affiliations:** 1 Stroke and Dementia Lab, “Luigi Sacco” Department of Biomedical and Clinical Sciences, University of Milan, Via Giovanni Battista Grassi 74, 20157 Milan, Italy; 2 Center for Cognitive Disorders and Dementias (CDCD), Neurology Unit, Ospedale Luigi Sacco, Milan, Italy

**Keywords:** BPSD, Dementia, Covid-19, SARS-CoV-2, Isolation, Mental health

## Abstract

**Background:**

During Covid-19 pandemic, the Italian government adopted restrictive limitations and declared a national lockdown on March 9, which lasted until May 4 and produced dramatic consequences on people’s lives. The aim of our study was to assess the impact of prolonged lockdown on behavioral and psychological symptoms of dementia (BPSD).

**Methods:**

Between April 30 and June 8, 2020, we interviewed with a telephone-based questionnaire the caregivers of the community-dwelling patients with dementia who had their follow-up visit scheduled from March 9 to May 15 and canceled due to lockdown. Among the information collected, patients’ BPSDs were assessed by the Neuropsychiatric Inventory (NPI). Non-parametric tests to compare differences between NPI scores over time and logistic regression models to explore the impact of different factors on BPSD worsening were performed.

**Results:**

A total of 109 visits were canceled and 94/109 caregivers completed the interview. Apathy, irritability, agitation and aggression, and depression were the most common neuropsychiatric symptoms experienced by patients both at baseline and during Covid-19 pandemic. Changes in total NPI and caregiver distress scores between baseline and during lockdown, although statistically significant, were overall modest. The logistic regression model failed to determine predictors of BPSD worsening during lockdown.

**Conclusion:**

This is one of the first studies to investigate the presence of BPSD during SARS-CoV-2 outbreak and related nationwide lockdown, showing only slight, likely not clinically relevant, differences in BPSD burden, concerning mostly agitation and aggression, anxiety, apathy and indifference, and irritability.

**Supplementary Information:**

The online version contains supplementary material available at 10.1007/s10072-020-05035-8.

## Introduction

Italy was the second country in the world to be hit by the SARS-CoV-2 outbreak, following in close time relation China. The first Italian Covid-19 case was reported in Codogno, a village in the Lombardia region, on February 21, 2020. Despite the attempt to lockdown a “red zone,” covering 11 small towns in the same area, SARS-CoV-2 rapidly spread throughout Italy, so that the government adopted restrictive limitations, including social distancing, and finally declared nationwide lockdown on March 9, which eventually lasted until May 4 [1].

As the Italian healthcare system risked to collapse due to the inconceivable number of patients admitted to emergency departments, with intensive care units gone almost saturated, the Italian Society of Anesthesia, Analgesia, and Intensive Care (SIAARTI) published ethics recommendations to face the critical issue of treatment allocation “in exceptional, resource-limited circumstances” [2]. Predictably, fatality rate was found higher in elderly people (as of June 22, 70–79 years: 26%; 80–89 years: 33.1%; ≥ 90 years: 31.3% in Italy), so that the age criterion, together with comorbidities, became pivotal when establishing how to employ extremely limited resources [3]. As a consequence, social distancing and isolation became critical for elderly people and, among them, for the vulnerable, broad group of patients affected by cognitive impairment.

Disruption of daily life represented an unforeseen stressful event for both younger and older people. It is worth noticing that the health status and health-related quality of life of older people is harshly affected by social isolation [4]. On March 18, the World Health Organization (WHO) published an interim guidance on “Mental health and psychosocial considerations during the COVID-19 outbreak”, highlighting the risks of social and physical isolation in elderly, especially in case of cognitive decline [5]. In this scenario, people with dementia and their caregivers had to face a dual challenge. On one side, they needed protection from an infection with high fatality rate among elderly patients; on the other, isolation and sudden changes of daily habits posed old subjects at high risk of mental health changes, requiring psychosocial and medical support that was however hard to reach during the Covid-19 pandemic. Contemporaneous to lockdown, the entire health system organization was heavily redesigned and many outpatients’ services, including dementia clinics, were closed [6].

People living with dementia presented an increased risk of exposure to Covid-19, because of the problems in remembering rules and regulations, and to the restricted access to public health information [7, 8]. To address this issue, both the WHO and Alzheimer Europe provided recommendations and advices for people with dementia, caregivers, and family members [5, 9]. Furthermore, behavioral and psychological symptoms of dementia (BPSDs), especially anxiety, agitation, loneliness, and depression, were expected to be increased during SARS-CoV-2 outbreak, possibly producing dramatic effects on physical and mental health [10]. In this scenario, Velayudhan et al. highlighted the risk of increased use of antipsychotics, hypnotics, and other sedatives in care home residents with neuropsychiatric conditions during SARS-CoV-2 outbreak to guarantee compliance with the restrictive measures adopted [10]. BPSDs are experienced by at least 98% of people living with dementia over the course of disease and are closely associated with both patient, caregiver, and environmental factors [11, 12]. Patient factors include undiagnosed acute medical illnesses [13]. Difficult access to care during Covid-19 pandemic may have increased the severity of unrelated medical conditions, raising concern about the consequences on population health produced by the measures adopted to limit SARS-CoV-2 spreading [14]. Regarding caregiver factors, instead, depression and anxiety were expected to be increased in formal and informal caregivers as a consequence of the Covid-19 risk perception and the high caregiver burden due to the unavailability of daily support services [15]. This can influence interaction between patient and caregiver, with a strong impact on neuropsychiatric symptoms of people with dementia [11]. Moreover, some BPSDs, such as agitation, are strongly influenced by exit control, one of the measures of social distancing and isolation adopted during Covid-19 pandemic [16]. Consistent with this evidence, previous studies highlighted the role of outdoor spaces, such as gardens, in decreasing agitation in care home residents with dementia [17].

Few works have addressed the issue of neuropsychiatric conditions associated with dementia during Covid-19 pandemic. Canevelli et al. described worsening or onset of BPSD in 54.7% of 139 patients with dementia and cognitive disturbances, specifically agitation and aggression, apathy, and depression [18]. A sub-study of an Italian multicenter nationwide survey showed increased BPSD burden in 59.6% patients. Irritability, apathy, agitation, and anxiety were the most frequently worsening symptoms during Covid-19 pandemic, whereas sleep disorder and irritability the main newly onset. One of the main limitations of both studies was represented by the lack of standardized assessment of BPSD through neuropsychiatric rating scales, and of data on previous BPSD severity or type [18, 19]. A systematic review, focused on neuropsychiatric symptoms of dementia during Covid-19 pandemic, revealed that apathy, anxiety, and agitation were the most frequent BPSD reported during SARS-CoV-2 outbreak, mainly triggered by prolonged isolation [20].

Starting from the above reported considerations, the aim of this study was to assess the impact of confinement on BPSD variations in community-dwelling patients affected by dementia. Furthermore, we explored the role of specific factors in BPSD development, worsening, or attenuation, including changes in living arrangements and lifestyle habits consequent to lockdown measures, access to outdoor spaces, and development of acute medical illness.

## Methods

### Design and data collection

The survey was conducted in the Center for Cognitive Disorders and Dementias (CDCD) of the “Luigi Sacco” Hospital in Milan. Two experienced neurologists contacted by phone caregivers of 109 community-dwelling adults affected by dementia who had a follow-up visit at the CDCD of the “Luigi Sacco” Hospital scheduled in the period from March 9 to May 15, and not carried out due to lockdown restriction of outpatients’ services. The call timeframe was from April 30 to June 8. The patients were affected by Alzheimer’s disease, frontotemporal dementia, dementia with Lewy bodies, corticobasal degeneration, vascular dementia, or mixed dementia, previously diagnosed by a dementia specialist of the CDCD, according to the current criteria [21–25]. Severity of dementia was categorized for this study based on Mini-Mental State Examination (MMSE) score (mild ≥ 20, moderate 15–20, moderately severe 11–14, severe ≤ 10).

During the telephone call, patients’ caregivers were interviewed with a semi-structured telephone-based questionnaire *ad hoc* developed by the authors, after obtaining oral informed consent. History and clinical data were collected from medical records.

The questionnaire (Supplementary Material) was composed of three sections:

1. Demographic characteristics of patient and caregiver; patient’s clinical data, living arrangements, and access to day care services before March 9;
2. Changes in accommodation, living arrangements and lifestyle habits due to lockdown measures; access to outdoor spaces from March 9 to May 4; access to emergency care and development of clinical signs or symptoms with or without need of hospitalization from March 9 to May 4;
3. Assessment of patient BPSD, including the Neuropsychiatric Inventory (NPI) full version [26], referring to the period before March 9, and to the period from March 9 to May 4; therapy adjustments and/or medical consults required to manage BPSD from March 9 to May 4.

The phone interviews lasted 30 min on the average.

### Data analysis

Statistical analyses were undertaken using open source software “Jamovi”, version 1.2 (Sidney, Australia). Participants’ characteristics are reported as means and standard deviation (SD) or frequencies (%). Since normality assumptions for parametric tests were not met according to Kolmogorov-Smirnov normality test, Wilcoxon signed-rank test was performed to compare differences between NPI scores over time. A *p*-value <0.05 was considered significant. Spearman’s rank correlation coefficient was used to test for correlation between NPI total score and NPI distress total score. After performing univariate binomial logistic regression analyses, a multivariate binomial logistic regression model was employed to analyze the effects of factors related to disease, caregiving, accommodation, and lockdown on BPSD worsening, defined as an increase in at least one NPI item frequency and/or severity score. Type and severity of dementia (MMSE category), baseline number of BPSD, caregiver type and gender, living arrangement, availability of social contacts, and access to outdoor spaces were inserted in the model as fixed effects, while age and sex as covariates.

## Results

### Participants

Among the 109 contacted caregivers, 11 refused to be surveyed and 4 were not interviewed as they reported that the patient had died in the period between the last outpatient visit and the phone call. A total of 94/109 caregivers successfully completed the interview (response rate = 86.2%).

### Socio-demographic and clinical data

The patients included in the study had a mean age of 83.2 years (SD 5.5), and 67/94 (71.3%) were women. Alzheimer’s disease was the most common diagnosis (83.0%). MMSE mean score was 17.5 (SD 6.1). Most patients (89.4%) had never attended day care services before March 9. Mean age of caregivers was 64.4 years (SD 14.7), and 64/94 (68.1%) were women. Socio-demographic characteristics and clinical data are summarized in Table 1.

**Table 1:** Sociodemographic characteristics, baseline clinical data and differences in accommodation, living arrangements, and lifestyle habits before and after SARS-CoV-2 outbreak

Characteristic	Total (*N* = 94)
Patient mean age in years ± SD	83.2 ± 5.5
Female patients, *n* (%)	71.3%
MMSE, mean ± SD (range)	17.5 ± 6.1 (0–27)
MMSE category, *n* (%)	
Mild (≥ 20)	33 (35.1%)
Moderate (15–20)	33 (35.1%)
Moderately severe (11–14)	19 (20.2%)
Severe (≤ 10)	9 (9.6%)
Diagnosis, *n* (%)	
Alzheimer’s disease	78 (83.0%)
Mixed dementia	7 (7.4%)
Vascular dementia	3 (3.2%)
Dementia with Lewy bodies	3 (3.2%)
Frontotemporal dementia	2 (2.1%)
Corticobasal degeneration	1 (1.1%)
Public and/or private day care services, *n* (%)	
None	84 (89.4%)
Day care center	8 (8.5%)
Other	2 (2.1%)
Caregiver, *n* (%)	
Spouse/partner	42 (44.7%)
Son/daughter	40 (42.6%)
Other – family member	7 (7.4%)
Other – not family member	5 (5.3%)
Caregiver mean age in years ± SD	64.4 ± 14.7
Female caregivers, *n* (%)	68.1%
Usual accommodation, *n* (%)	
Own house	88 (93.6%)
Caregiver’s house	5 (5.3%)
Other	1 (1.1%)
Usual living arrangement, *n* (%)	
None	17 (18.1%)
Unlicensed assistive personnel	27 (28.7%)
Caregiver	55 (58.5%)
Other family member/s	19 (20.2%)
Patient change of accommodation during SARS-CoV-2 outbreak, *n* (%)	6 (6.4%)
Number of cohabitants during SARS-CoV-2 outbreak, *n* (%)	
None	4 (4.3%)
1	63 (67.0%)
≥ 2	27 (28.7%)
Visits of family members during SARS-CoV-2 outbreak, *n* (%)	72 (76.6%)
Access to outdoor spaces during SARS-CoV-2 outbreak, *n* (%)	22 (23.4%)

Data are mean ± SD, or *n* (%)

### Accommodation, living arrangements, and lifestyle habits

None of the patients enrolled in the study was living in long-term care facilities, nursing homes, or hospices before March 9, and 77/94 (81.9%) lived accompanied. Within the group of patients living accompanied, 27/77 (35.1%) were living with unlicensed assistive personnel (UAP). After SARS-CoV-2 outbreak, 6/27 (22.2%) UAPs had to leave the previous accommodation with the patient. Of these, 2 patients moved to caregiver’s home, 3 continued living with their caregiver, and only 1 was left alone. Only 1/94 (1.1%) caregiver moved to patient’s house, while 6/94 (6.4%) patients had to change house. As aforementioned, 2 of them belonged to the group of patients who separated from their UAPs during lockdown; 3 were living alone before March 9 and moved to caregiver’s home during lockdown; 1 continued living with the caregiver, but they changed house for personal reasons. Four out of 17 (23.5%) patients not living accompanied before SARS-CoV-2 outbreak continued living alone during lockdown. The remaining 13 (76.5%) patients began living with a UAP during lockdown. Most patients (76.6%) continued receiving visits from family members during SARS-CoV-2 outbreak. Only a minority (23.4%) performed regular outdoor walking during lockdown, in accordance with government regulations for people with physical and cognitive disability.

Data about accommodation, living arrangements, and lifestyle habits before and after SARS-CoV-2 outbreak are reported in Table 1.

### Access to emergency care and development of new clinical signs or symptoms

From March 9 to May 15, 4/94 (4.3%) patients were admitted to hospital. In all 4 cases, the cause of hospital admission was Covid-19. Fourteen out of 94 (14.9%) patients developed clinical symptoms or disturbances with no need of hospitalization, including pain, fever, malaise, cough, diarrhea, vomit, asthenia, hypertensive crisis, and urinary tract infection.

### BPSD

Only 7/94 (7.4%) participants were free of BPSD before March 9. Apathy was the most common neuropsychiatric symptom experienced by patients both at baseline (45.8%) and during Covid-19 pandemic (51.1%), followed by irritability (41.5% and 42.6%), agitation and aggression (35.1% and 38.3%), and depression (30.9% and 31.9%).

Only four items showed new onset or, more frequently, a worsening of either frequency, severity, or both in at least 10% of subjects, specifically agitation and aggression (21.3%), anxiety (14.9%), apathy and indifference (12.8%), and irritability (11.7%) (Table 2). A total of 20/94 (21.3%) patients had an increased NPI score of only one item after March 9, whereas 30/94 (31.9%) of two or more.

**Table 2:** Percentage of patients who developed new BPSD or had worsening of pre-existing BPSD during lockdown

	Patients with new symptoms during lockdown (%)	Patients with worsening of pre-existing symptoms during lockdown (%)
Delusions	4.3%	2.1%
Hallucinations	3.2%	3.2%
Agitation – Aggression	4.3%	17.0%
Depression	1.1%	8.5%
Anxiety	9.6%	5.3%
Elation – Euphoria	0%	0%
Apathy – Indifference	4.3%	8.5%
Disinhibition	1.1%	0%
Irritability	2.1%	9.6%
Aberrant motor behavior	0%	6.4%
Sleep and nighttime behavior disorders	4.3%	2.1%
Appetite and eating disorders	2.1%	1.1%

*BPSD*, behavioral and psychological symptoms of dementia

**Fig. 1:**
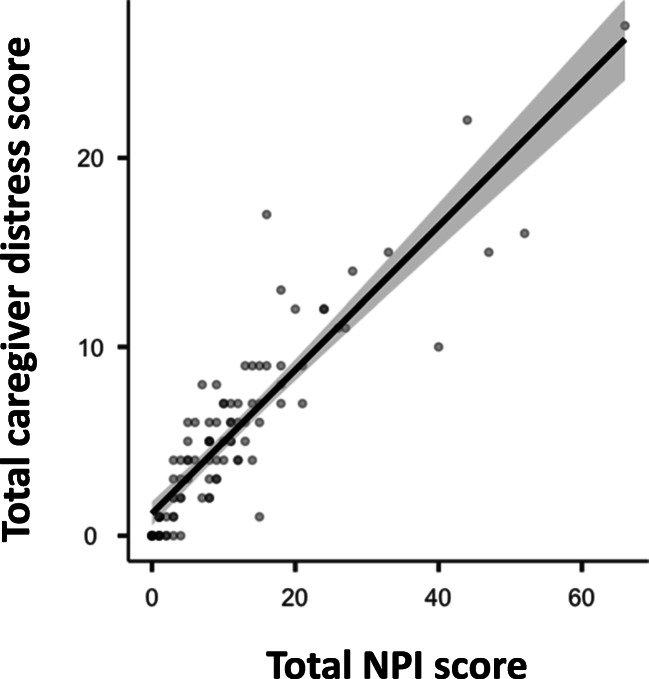
Scatterplot graph of the association between total NPI score and total caregiver distress score

Mean total NPI score before March 9 was 9.0 (SD 5.0), whereas the caregiver distress scale showed a mean score of 4.5 (SD 3.0). They respectively increased to 11.5 (9.0) and 5.5 (5.0) during nationwide lockdown (Table 3). A statistically significant correlation between total NPI score and total caregiver distress score was found both at baseline (rho = 0.877, *p*-value <0.001) and during SARS-CoV-2 outbreak (rho = 0.893, *p-*value <0.001) (Fig. 1). The increase of total NPI score and total caregiver distress score between baseline and the lockdown was statistically significant (*p-*value <0.001) but overall modest. Their mean variations (respectively 2.5 and 1.0 points) were slight, especially if compared with the far larger ranges of total NPI score and total caregiver distress score (respectively 0–144 and 0–60 points) (Table 3).

**Table 3:** Comparison of BPSD, assessed using NPI full version, between baseline and after SARS-CoV-2 outbreak

	Before lockdown	During lockdown	Wilcoxon signed-rank test	*p-*value
Total NPI score				
Mean (SD)	9.0 (5.0)	11.5 (9.0)	306.0	**<0.001**
Range	0–63	0–66	-	-
Total NPI score–single items, mean (SD)				
Delusions	0.3 (1.3)	0.4 (1.4)	9.0	0.23
Hallucinations	0.2 (0.8)	0.3 (1.3)	5.5	0.17
Agitation – Aggression	1.4 (2.9)	2.0 (3.3)	53.0	**0.003**
Depression	1.2 (2.6)	1.4 (3.1)	16.0	0.14
Anxiety	0.5 (1.7)	0.8 (2.0)	27.5	**0.018**
Elation – Euphoria	0.0 (0.4)	0.1 (0.9)	0.0	1.00
Apathy – Indifference	1.8 (2.6)	2.2 (2.9)	3.0	**0.003**
Disinhibition	0.1 (0.5)	0.1 (0.6)	6.0	0.85
Irritability	1.4 (2.4)	1.7 (2.8)	17.5	**0.029**
Aberrant motor behavior	0.5 (2.0)	0.7 (2.3)	4.0	0.11
Sleep and nighttime behavior disorders	1.0 (2.8)	1.2 (3.0)	4.5	0.25
Appetite and eating disorders	0.5 (1.3)	0.6 (1.7)	− 1.3	0.20
Total caregiver distress score				
Mean (SD)	4.5 (3.0)	5.5 (5.0)	214.0	**<0.001**
Range	0–28	0–27	-	-
Caregiver distress score–single items, mean (SD)				
Delusions	0.2 (0.7)	0.3 (0.7)	11.0	0.66
Hallucinations	0.1 (0.5)	0.2 (0.6)	3.5	0.71
Agitation – Aggression	0.8 (1.2)	1.0 (1.5)	22.5	**<0.001**
Depression	0.5 (0.9)	0.5 (1.1)	4.0	0.41
Anxiety	0.3 (0.7)	0.5 (1.0)	15.5	**0.011**
Elation – Euphoria	0.0 (0.3)	0.0 (0.3)	0.0	1.00
Apathy – Indifference	0.8 (1.0)	0.9 (1.1)	0.0	**0.018**
Disinhibition	0.1 (0.4)	0.1 (0.5)	6.0	0.86
Irritability	0.9 (1.2)	1.0 (1.3)	29.0	0.13
Aberrant motor behavior	0.3 (0.9)	0.3 (1.0)	3.0	0.12
Sleep and nighttime behavior disorders	0.4 (1.1)	0.6 (1.3)	7.5	0.08
Appetite and eating disorders	0.2 (0.6)	0.3 (0.7)	5.0	0.28
NPI items worsening (frequency, severity or both), *n* (%)				
0	NA	44 (46.8%)	-	-
1	NA	20 (21.3%)	-	-
> 1	NA	30 (31.9%)	-	-

Data are mean (SD) or *n* (%). Significant associations (*p*-value <0.05) are represented in bold. *BPSD*, behavioral and psychological symptoms of dementia; *NPI*, Neuropsychiatric Inventory

Similarly, comparative analysis of total score for each NPI item showed a statistically significant increase in NPI score related to agitation and aggression (*p-*value 0.003), anxiety (*p-*value 0.018), apathy and indifference (*p-*value 0.003), and irritability (*p-*value 0.029) during lockdown, as well as in caregiver distress score of agitation and aggression (*p-* value <0.001), anxiety (*p-*value 0.011), and apathy and indifference (*p-*value 0.018). However, the mean changes from before March 9 and lockdown period were small (NPI score of agitation and aggression: 0.6 points, anxiety: 0.3 points, apathy and indifference: 0.4 points, irritability: 0.3 points; caregiver distress score of agitation and aggression: 0.2 points, anxiety 0.2 points, apathy and indifference 0.1 points) (Table 3).

Univariate binomial logistic regression analyses were performed in order to evaluate the effects of possible predictors of BPSD worsening independently of each other, and no significant relations were found. Nevertheless, in order to estimate if the combination of factors could highlight any possible effect of predictors, a multivariate model was built. The model failed to determine predictors of BPSD worsening during lockdown, including type and severity of dementia (MMSE category), baseline number of BPSD, caregiver type and gender, living arrangement, availability of social contacts, and access to outdoor spaces during SARS-CoV-2 outbreak (Table 4).

**Table 4:** Predictors of BPSD worsening, corresponding to an increase in at least one NPI item frequency and/or severity score, after SARS-CoV-2 outbreak

Predictor	*p-*value	OR (95% CI)
Baseline number of BPSD (ref: absence of BPSD)		
1	0.462	2.2 (0.3–17.3)
> 1	0.116	5.1 (0.7–38.5)
Type of dementia (ref: AD)		
Other	0.053	0.3 (0.1–1.0)
Severity of dementia (ref: severe–MMSE ≤10)		
Moderately severe (MMSE 11–14)	0.362	2.3 (0.4–13.8)
Moderate (MMSE 15–20)	0.419	0.5 (0.1–2.6)
Mild (MMSE >20)	0.845	0.8 (0.2–4.7)
Caregiver (ref: spouse–partner)		
Son–daughter	0.641	1.4 (0.4–4.9)
Other	0.734	1.3 (0.3–5.7)
Caregiver gender (ref: male)		
Female	0.680	0.8 (0.2–2.8)
Living arrangement (ref: 0)		
1	0.186	0.2 (0.0–2.5)
> 1	0.390	0.3 (0.2–5.1)
Visits of family members during SARS-CoV-2 outbreak (ref: yes)		
No	0.652	1.3 (0.4–4.1)
Access to outdoor spaces during SARS-CoV-2 outbreak (ref: yes)		
No	0.796	1.2 (0.4–3.6)

*Covariates (age, sex) did not modify the models results. *OR*, odds ratio; *CI*, confidence interval; *BPSD*, behavioral and psychological symptoms of dementia; *NPI*, Neuropsychiatric Inventory

Twenty-two out of ninety-four (23.4%) patients required medical phone consults to manage BPSD, and 14/94 (14.9%) needed therapeutic adjustments (9.6% received an increased dosage of medications already used, whereas 5.3% started a new treatment). The therapeutic approach included mostly antipsychotic (quetiapine 6.4%; promazine 2.1%; haloperidol 1.1%) and antidepressant drugs (trazodone 4.3%; mirtazapine 1.1%).

## Discussion

Our study investigated the natural history of BPSD during SARS-CoV-2 outbreak and related nationwide lockdown. The cohort of patients with dementia enrolled in the study overall showed moderate BPSD burden at baseline, however with high variability. During lockdown, we found that patients experienced only slightly increased total BPSD burden, likely not clinically relevant. It is worth noticing that the moderate BPSD burden at baseline might have influenced the poor results emerged from the study. Apathy was the most common neuropsychiatric symptom in our cohort both at baseline and during Covid-19 pandemic, followed by irritability, agitation, and aggression, and depression. Furthermore, the variations of NPI score related to agitation and aggression, anxiety, apathy and indifference, and irritability during lockdown, although statistically significant, were so modest that their clinical relevance is doubtful. As a consequence, the results of our study do not allow drawing definite conclusions regarding the role of the Covid-19 pandemic in neuropsychiatric deterioration of patients affected by dementia.

Similarly to total NPI score, total caregiver distress score appeared barely increased compared to baseline. It should also be considered that, given the exceptional circumstance of Covid-19 pandemic, caregiver distress might have been caused not only by patients BPSD but also by the impact of the lockdown itself on the general population. However, it is worth noticing that total NPI score and total caregiver distress score were strictly correlated. Therefore, we are not able to exclude a role of the variations in total patient BPSD burden during lockdown in the slightly increased caregiver distress.

In most cases, we detected worsening of pre-existing symptoms rather than development of additional ones, suggesting that home confinement during SARS-CoV-2 outbreak might have played a role especially on pre-existing neuropsychiatric symptoms. Unfortunately, our logistic regression model failed to determine predictors of change in total NPI score and total caregiver distress score, so that the specific effects of different factors related to disease, caregiving, accommodation, and home confinement on BPSD development or worsening remain uncovered.

This survey recorded also four subjects admitted to hospital for Covid-19 infection. A previous work highlighted the presence of delirium as the most common symptom at onset in patients affected by dementia [27]. Nevertheless, no clues for differences in behavior, possibly indicating delirium, emerged in our small sample of patients with SARS-CoV-2 infection compared to the remaining subjects, even though the limited number of cases does not allow drawing conclusions. An alternative explanation might be the higher prevalence of hypoactive form of delirium among Covid-19 patients with dementia, which is often not recognized [27].

Our study presents limitations. First, retrospective assessment of BPSDs carried out during Covid-19 pandemic might have introduced biases, particularly related to the distress underwent by the general population, including caregivers, during lockdown. However, the unexpected outbreak of Covid-19 pandemics made this approach difficult to avoid. Furthermore, our goal was to determine possible changes of BPSD burden introduced by the lockdown measures. To achieve this aim, we needed a BPSD measurement referred to the period immediately before lockdown, as more antecedent assessments would have not been able to distinguish variations strictly associated with Covid-19 pandemics from those occurring as part of the natural history of the disease. Second, this study lacks a control group, which would have been helpful in defining if the detected small changes were associated with home confinement during SARS-CoV-2 outbreak or if they merely represented the foreseeable progression of the disease. Third, we need to outline that the psychological effects of lockdown on the general population remain unclear and this knowledge would be required to assess the net effect of these restrictions on our patients as well as on caregivers. Finally, the telephone interview allowed collecting only a restricted amount of data. Nevertheless, this type of contact was obliged during the nationwide lockdown, was the same method of other studies on the same topic [18, 19], and remained the safest one also after May 4, especially in case of vulnerable patients affected by dementia.

The main strength of this study, which differentiates it from other works that have addressed the issue of neuropsychiatric conditions associated with dementia during Covid-19 pandemic [18, 19], is represented by the use of a standardized assessment of BPSD through the NPI, a neuropsychiatric rating scale commonly employed worldwide. Our work revealed worsening or onset of BPSD in a percentage of patients (53.2%) comparable with those emerged in previous studies [18, 19]. However, the use of the NPI allowed us to quantify the BPSD change, thus revealing only a slight increase, likely not clinically relevant, which represents an added value of our investigation.

This work offers the opportunity to explore the overall BPSD burden during home confinement, even though the circumstance of analysis was exceptional. The changes detected in neuropsychiatric symptoms were overall modest and of unclear clinical relevance, suggesting that people with dementia present a fairly large resilience, and that BPSD may not be strongly influenced by quarantine and changes in daily life at least when they are time limited. Nonetheless, further studies in different conditions are required to guide physicians in all the circumstances that might alter routine life of dementia patients, in order to prevent behavioral and psychiatric deterioration and guarantee continued access to care despite the limitations imposed by their vulnerability.

## Supplementary Information

Supplementary File 1 – Telephone based-survey employed for the study (DOCX 36 kb).

## Data Availability

The datasets generated for this study will not be made publicly available due to privacy. Nevertheless, further analyses might be available from authors by request to the corresponding author.
