# Supplementary material for: The impact of lockdown during SARS-CoV-2 outbreak on behavioral and psychological symptoms of dementia
Source: Neurol Sci. 2021 Jan 14;42(3):825–33. doi: 10.1007/s10072-020-05035-8 (PMC7806279; doi:10.1007/s10072-020-05035-8)
Supplement: Supplementary file 1 — – Telephone based-survey employed for the study (DOCX 36 kb). [file 10072_2020_5035_MOESM1_ESM.docx]

***The impact of lockdown measures during SARS-CoV-2 outbreak on behavioral and psychological symptoms of dementia: a telephone-based survey***

*“Luigi Sacco” Hospital of Milan*

*Center for Cognitive Disorders and Dementias (CDCD)*

**SECTION I – Demographic characteristics of patient and caregiver; patient’s clinical data, living arrangements and access to day care services before March 9**

1. Patient initials:

______

1. Patient date of birth:

______

1. Reference number:

______

1. Patient gender:
   1. Male
   2. Female
2. Patient diagnosis:
   1. Alzheimer’s disease
   2. Frontotemporal dementia
   3. Dementia with Lewy Bodies
   4. Corticobasal degeneration
   5. Vascular dementia
   6. Mixed dementia
   7. Other (specify):

______________________________________________________________________________

______________________________________________________________________________

1. Patient’s last MMSE - score:

______

1. Patient’s last MMSE – date:

______

1. Information gathered from:
   1. Spouse/partner
   2. Son/daughter
   3. Other family member
   4. In-home nurse
   5. Other (specify):

______________________________________________________________________________

______________________________________________________________________________

1. Who is the patient’s caregiver?
   1. Spouse/partner
   2. Son/daughter
   3. Other (family member) - specify:

______________________________________________________________________________

- 1. Other (not family member) - specify:

______________________________________________________________________________

1. Caregiver age:

____________________________________________________________________________________

1. Caregiver gender:
   1. Male
   2. Female
2. Where did the patient live before March 9?
   1. Own house
   2. Caregiver’s house
   3. Other family member’s house
   4. Other house (not family member)
   5. Hospital
   6. Nursing home or long-term care facility
   7. Hospice
   8. Rehabilitation center
   9. Other (specify):

______________________________________________________________________________

______________________________________________________________________________

1. Who did the patient live with before March 9? (You can choose more than one answer)
   1. None
   2. In-home nurse
   3. Caregiver
   4. Other family member/s
   5. Other (specify):

______________________________________________________________________________

______________________________________________________________________________

1. What public and/or private day care services did the patient attend before March 9^th^? (You can choose more than one answer)
   1. Day care center (the Italian “Centri Diurni Integrati” or CDI)
   2. Centers of Psychology for elderly and Alzheimer’s disease (the Italian “Centri di Psicologia per l’Anziano e l’Alzheimer” or CPAA)
   3. Alzheimer Cafè
   4. Psychosocial intervention
   5. Did not attend any day care services
   6. Other (specify):

______________________________________________________________________________

______________________________________________________________________________

1. How often did the patient attend public and/or private day care services before March 9?
   1. Everyday
   2. Weekly (once or twice a week)
   3. Monthly (once or twice a month)
   4. Sporadically
   5. Did not attend any day care services
2. What public and/or private day care services did the caregiver/other family member attend before March 9? (You can choose more than one answer)
   1. Centers of Psychology for elderly and Alzheimer’s disease (the Italian “Centri di Psicologia per l’Anziano e l’Alzheimer” or CPAA)
   2. Alzheimer Cafè
   3. Did not attend any day care service
   4. Other (specify):

______________________________________________________________________________

______________________________________________________________________________

1. How often did the caregiver/other family member attend public and/or private day care services before March 9? (You can choose more than one answer)
   1. Everyday
   2. Weekly (once or twice a week)
   3. Monthly (once or twice a month)
   4. Sporadically
   5. Did not attend any day care service

**SECTION II – Changes in living arrangements and lifestyle habits due to lockdown measures, access to outdoor spaces, access to emergency care and development of clinical signs or symptoms with or without need to hospitalization from March 9 to May 4**

1. Did the patient move to another domicile after March 9?
   1. Yes
   2. No
2. What are the dimensions of the current domicile compared to the usual one?
   1. Same
   2. Higher
   3. Smaller
   4. Did not move to another domicile
3. Has the patient kept meeting family members that he/she usually met before March 9?
   1. Yes
   2. No
4. Did the caregiver/other family member move to the patient’s domicile after March 9?
   1. Yes
   2. No
5. Did the patient live with an in-home nurse who had to move away after March 9?
   1. Yes
   2. No
6. How many people does the patient currently live with?
   1. 1
   2. 2-4
   3. ≥5
7. Does the house where the patient currently lives have a garden?
   1. Yes
   2. No
8. Has the patient accessed to emergency care or got hospitalized from March 9 to May 4 for BPSD?
   1. Yes
   2. No
9. Has the patient accessed to emergency care or got hospitalized from March 9 to May 4 for SARS-CoV-2 infection?
   1. Yes
   2. No
10. Has the patient accessed to emergency care or got hospitalized from March 9 to May 4 for other signs and/or symptoms (not BPSD, not SARS-CoV-2 infection)?
    1. Yes
    2. No
11. Has the patient developed clinical signs and/or symptoms without need to hospitalization from March 9 to May 4?
    1. Yes
    2. No
12. If yes: which signs and/or symptoms?

____________________________________________________________________________________________________________________________________________________________

1. Has the patient enjoyed outdoor spaces, together with the caregiver or other family member, from March 9 to May 4?
   1. Yes
   2. No

**SECTION III – Assessment of patient BPSD, including the Neuropsychiatric Inventory (NPI), before March 9, and from March 9 to May 4; therapeutic adjustments and/or medical consults required to manage BPSD from March 9 to May 4**

1. Assessment of the Neuropsychiatric Inventory (NPI) before March 9:

|  | Before March 9 | | | | | | |
| --- | --- | --- | --- | --- | --- | --- | --- |
|  | YES | NO | NA | Frequency | Severity | Total | Caregiver distress |
| Delusions |  |  |  |  |  |  |  |
| Hallucinations |  |  |  |  |  |  |  |
| Agitation / Aggression |  |  |  |  |  |  |  |
| Depression |  |  |  |  |  |  |  |
| Anxiety |  |  |  |  |  |  |  |
| Elation / Euphoria |  |  |  |  |  |  |  |
| Apathy / Indifference |  |  |  |  |  |  |  |
| Disinhibition |  |  |  |  |  |  |  |
| Irritability |  |  |  |  |  |  |  |
| Aberrant motor behavior |  |  |  |  |  |  |  |
| Sleep and nighttime behavior disorders |  |  |  |  |  |  |  |
| Appetite and eating disorder |  |  |  |  |  |  |  |
| TOTAL NPI SCORE |  |  |  |  |  |  |  |
| TOTAL DISTRESS SCORE |  |  |  |  |  |  |  |

1. Assessment of the Neuropsychiatric Inventory (NPI) from March 9 to May 4:

|  | From March 9 to May 4 | | | | | | |
| --- | --- | --- | --- | --- | --- | --- | --- |
|  | YES | NO | NA | Frequency | Severity | Total | Caregiver distress |
| Delusions |  |  |  |  |  |  |  |
| Hallucinations |  |  |  |  |  |  |  |
| Agitation / Aggression |  |  |  |  |  |  |  |
| Depression |  |  |  |  |  |  |  |
| Anxiety |  |  |  |  |  |  |  |
| Elation / Euphoria |  |  |  |  |  |  |  |
| Apathy / Indifference |  |  |  |  |  |  |  |
| Disinhibition |  |  |  |  |  |  |  |
| Irritability |  |  |  |  |  |  |  |
| Aberrant motor behavior |  |  |  |  |  |  |  |
| Sleep and nightime behavior disorders |  |  |  |  |  |  |  |
| Appetite and eating disorder |  |  |  |  |  |  |  |
| TOTAL NPI SCORE |  |  |  |  |  |  |  |
| TOTAL DISTRESS SCORE |  |  |  |  |  |  |  |

1. Has the patient required medical consults for the management of BPSD from March 9 to May 4?
   1. Yes
   2. No
2. Has the patient required a dosage increase of usual medications for the management of BPSD from March 9 to May 4?
   1. Yes (specify):

____________________________________________________________________________________________________________________________________________________________

- 1. No

1. Has the patient required the employment of medications not used before for the management of BPSD from March 9 to May 4?
   1. Yes (specify):

____________________________________________________________________________________________________________________________________________________________

- 1. No

1. Did the dosage increase of usual medications or the employment of medications not used before produce side effects?
   1. Yes (specify):

____________________________________________________________________________________________________________________________________________________________

- 1. No
  2. Did not require dosage increase of usual medications or the employment of medications not used before

1. What public counselling services did the patient attend from March 9 to May 4? (You can choose more than one answer)
   1. Specific phone lines
   2. Psychology Centers with services employing calls and/or videocalls
   3. Did not attend any public counselling service
   4. Other (specify): ______________________________________________________________________________

______________________________________________________________________________

1. How often did the patient attend public counselling services from March 9 to May 4?
   1. Once from March 9
   2. Once a week from March 9
   3. More than once a week from March 9
   4. Did not attend any public counselling service
2. What was the impact of public counselling services on the management of BPSD from March 9 to May 4?
   1. Absent
   2. Minimal
   3. Mild
   4. Moderate
   5. Evident
   6. Did not attend any public counselling service
